# Meta-analysis of structural and functional brain abnormalities in early-onset schizophrenia

**DOI:** 10.3389/fpsyt.2024.1465758

**Published:** 2024-08-23

**Authors:** Lu Wang, Ruishan Liu, Juan Liao, Xin Xiong, Linfeng Xia, Weiwei Wang, Junqi Liu, Fulin Zhao, Lihua Zhuo, Hongwei Li

**Affiliations:** ^1^ Medical Imaging College, North Sichuan Medical College, Nanchong, China; ^2^ Department of Radiology, The Third Hospital of Mianyang, Sichuan Mental Health Center, Mianyang, China; ^3^ Department of Neurosurgery, The Third Hospital of Mianyang, Sichuan Mental Health Center, Mianyang, China; ^4^ Department of Psychiatry, The Third Hospital of Mianyang, Sichuan Mental Health Center, Mianyang, China

**Keywords:** early-onset schizophrenia, voxel-based morphometry, gray matter volume, resting state functional imaging, multimodal, meta-analysis

## Abstract

**Background:**

Previous studies based on resting-state functional magnetic resonance imaging(rs-fMRI) and voxel-based morphometry (VBM) have demonstrated significant abnormalities in brain structure and resting-state functional brain activity in patients with early-onset schizophrenia (EOS), compared with healthy controls (HCs), and these alterations were closely related to the pathogenesis of EOS. However, previous studies suffer from the limitations of small sample sizes and high heterogeneity of results. Therefore, the present study aimed to effectively integrate previous studies to identify common and specific brain functional and structural abnormalities in patients with EOS.

**Methods:**

The PubMed, Web of Science, Embase, Chinese National Knowledge Infrastructure (CNKI), and WanFang databases were systematically searched to identify publications on abnormalities in resting-state regional functional brain activity and gray matter volume (GMV) in patients with EOS. Then, we utilized the Seed-based *d* Mapping with Permutation of Subject Images (SDM-PSI) software to conduct a whole-brain voxel meta-analysis of VBM and rs-fMRI studies, respectively, and followed by multimodal overlapping on this basis to comprehensively identify brain structural and functional abnormalities in patients with EOS.

**Results:**

A total of 27 original studies (28 datasets) were included in the present meta-analysis, including 12 studies (13 datasets) related to resting-state functional brain activity (496 EOS patients, 395 HCs) and 15 studies (15 datasets) related to GMV (458 EOS patients, 531 HCs). Overall, in the functional meta-analysis, patients with EOS showed significantly increased resting-state functional brain activity in the left middle frontal gyrus (extending to the triangular part of the left inferior frontal gyrus) and the right caudate nucleus. On the other hand, in the structural meta-analysis, patients with EOS showed significantly decreased GMV in the right superior temporal gyrus (extending to the right rolandic operculum), the right middle temporal gyrus, and the temporal pole (superior temporal gyrus).

**Conclusion:**

This meta-analysis revealed that some regions in the EOS exhibited significant structural or functional abnormalities, such as the temporal gyri, prefrontal cortex, and striatum. These findings may help deepen our understanding of the underlying pathophysiological mechanisms of EOS and provide potential biomarkers for the diagnosis or treatment of EOS.

## Introduction

Schizophrenia is a severe psychiatric disorder characterized by high disabling and progressive development, and the main clinical symptoms are positive symptoms (e.g. hallucinations, delusions, etc.), negative symptoms (e.g. affective apathy, impoverished thinking, etc.), and cognitive impairment (1). The World Health Organization (WHO) reported that schizophrenia affected approximately 24 million people or 1 in 300 people (0.32%) worldwide in 2022 (2). Schizophrenia has become a major public health problem worldwide, causing serious harm to patients and their families, as well as a heavy economic burden on society and government. Specifically, compared with the general population, patients with schizophrenia have a 2.08 times increased risk of death (3), and their mean life expectancy is shortened by about 15 years (4). On the other hand, patients with schizophrenia often suffer from social exclusion and human rights violations, which may extend to their family members (5). In addition, the combined direct and indirect economic burden of schizophrenia in the United States exceeded $340 billion (6). As mentioned above, the dangers of schizophrenia are obvious, over the past decades, despite extensive basic and clinical studies on schizophrenia by many researchers, the pathophysiological mechanisms of schizophrenia remain unclear, and effective treatments are still lacking. In response to the above problems, many researchers have put forward different hypotheses in an attempt to reveal the pathophysiological mechanisms of schizophrenia, among which the widely accepted view is that schizophrenia is related to alterations in the neural developmental trajectory of the brain due to abnormalities in the action of genetic, environmental and other factors (7). Early-onset schizophrenia (EOS) is a subgroup of schizophrenia that is defined as the first onset of symptoms of schizophrenia before the age of 18. EOS accounts for approximately 5% of all schizophrenia cases and is characterized by higher genetic susceptibility, more atypical and severe symptomatology, and poorer therapeutic response to antipsychotic medications compared with adult-onset schizophrenia (8–10). Notably, EOS is less affected by potential confounders such as antipsychotic medications, life events, and the social environment (11). Therefore, conducting studies on patients with EOS provides a unique perspective on schizophrenia research and may be helpful in the exploration of the pathophysiological mechanisms of schizophrenia.

With the development of neuroimaging techniques, resting-state functional magnetic resonance imaging (rs-fMRI) and voxel-based morphometry (VBM) have been widely used in the studies of functional and structural brain abnormalities in patients with EOS. Rs-fMRI can be used to detect the spontaneous activity of neurons in the brain when subjects are not performing any specific task or receiving any external stimulation, and it can effectively avoid instability caused by subjects performing specific tasks (12–14). Hence, rs-fMRI provides a powerful tool for the exploration of resting-state regional functional brain activity in patients with EOS. The alterations of resting-state regional functional brain activity can be represented by indicators such as amplitude of low-frequency fluctuation (ALFF), fractional ALFF (fALFF), regional homogeneity (ReHo) and cerebral blood flow (CBF). Among which, ALFF indirectly reflects regional functional brain activity by measuring the total power of blood oxygenation level-dependent (BOLD) signals in the low frequency range (15). fALFF reflects the relative contribution of oscillations in the low frequency range to the overall detectable frequency range (16). ReHo is an assessment of the synchronization between a given voxel and a neighboring voxel time-series through the consistency of the Kendall’s coefficient of concordance (17). In addition, CBF can be quantified by fMRI, single-photon emission computed tomography (SPECT) and positron emission tomography (PET) using the arterial spin labelling (ASL) technique (18). Previous studies based on the above methodologies had identified significant abnormalities in resting-state regional functional brain activity in patients with EOS versus healthy controls (HCs). However, there was high heterogeneity in these results. For example, some studies have reported significantly increased resting-state regional functional brain activity in prefrontal cortex (PFC) in patients with EOS (19–21), however, other studies have observed significant decreased resting-state functional activity in PFC (22, 23). In addition, it has been identified that patients with EOS have both regions of increased and decreased resting-state regional functional brain activity in the PFC (24).

In addition to the abnormalities in resting-state functional brain activity, previous studies had revealed significant anatomical abnormalities in patients with EOS.VBM is an automated whole-brain technique capable of assessing regional grey matter volume (GMV) alterations without bias (25). GMV, as a structural marker that is relatively stable over time, can reflect to a certain extent the structural basis behind alterations in functional brain activity (26). Similarly, there were discrepancies in the results of the VBM studies. For instance, some previous VBM studies shown decreased regional GMV in the inferior temporal gyrus in patients with EOS (27–29), yet another study reported increased GMV in the inferior temporal gyrus in patients with EOS (30). The inconsistency in the results of the VBM and rs-fMRI studies described above may be attributed to the limited sample size of the studies, the heterogeneity of the study methodology (e.g., different inclusion and exclusion criteria, different methods of data analysis, etc.), and the heterogeneity of the study subjects (e.g., different duration of the disease, severity of the disease, and medication status, etc.).

Hence, the present study aimed to explore the most reliable and consistent structural and functional brain abnormalities in EOS by systematically reviewing and effectively integrating previous studies on resting-state regional functional brain activity and GMV conducted on EOS patients. Alterations in functional brain activity and structural abnormalities are closely related, so another aim of the present study was to explore whether there is a corresponding structural basis for brain regions with abnormal resting-state functional brain activity in patients with EOS. In brief, the present study began with separate meta-analysis of rs-fMRI studies and VBM studies, followed by multimodal overlapping on this basis. We then conducted a validation subgroup meta-analysis after excluding uncorrected for statistics to test the robustness of the results of the main meta-analyses. We also performed subgroup meta-analyses of the rs-fMRI studies and the VBM studies, respectively, to investigate whether there are different functional and structural abnormalities in patients with EOS in different disease statuses. Specifically, two subgroup meta-analyses were included (studies of patients with first-episode EOS and studies of patients with drug-naive EOS). Finally, we conducted meta-regression analyses to explore potential associations of clinical variables (age, sex, years of education, duration of illness, and symptom severity scores) with the results of the main meta-analyses. Based on the evidence from previous studies, we speculated that the brain regions in which EOS patients develop abnormalities in GMV and resting-state regional functional brain activity are mainly located in the PFC, temporal gyrus and striatum.

## Methods

### Literature search

This study followed the Preferred Reporting Items for Systematic Reviews and Meta-Analyses (PRISMA) guidelines (31). The current meta-analysis was registered with PROSPERO (registration number: CRD42024544361). In the present study, we conducted a systematic and comprehensive search for studies on VBM and rs-fMRI in patients with EOS published in PubMed, Web of Science, Embase, Chinese National Knowledge Infrastructure (CNKI) and WanFang databases through April 30, 2024, combined with the following keywords:(“schizophrenia” OR “schizophrenics” OR “schizophrenic disorder”) AND (“functional magnetic resonance imaging” OR “fMRI” OR “resting-state” OR “amplitude of low-frequency fluctuation” OR “ALFF” OR “fractional ALFF” OR “fALFF” OR “regional homogeneity” OR “ReHo” OR “cerebral blood flow” OR “CBF” OR “positron emission tomography” OR “PET” OR “single photon emission computed tomography” OR “SPECT” OR “arterial spin labeling” OR “ASL” OR “voxel-based morphometry” OR “gray matter” OR “VBM”) AND (“adolescent” OR “child” OR “early-onset”). In addition, the references of the included studies and relevant review literature were examined to avoid the omission of other relevant studies.

### Study selection

Studies were included in the meta-analysis if they satisfied the following criteria: (1) the study was original (rather than a review or abstract, etc.) that was peer-reviewed for publication in English or Chinese language journal; (2) the study subjects were formally diagnosed with schizophrenia before the age of 18 according to DSM, ICD or other criteria; (3) they analyzed resting-state functional brain activity or GMV at the whole-brain level; (4) they compared regional resting-state functional brain activity or GMV between patients with EOS and HCs; (5) peak coordinates based on whole-brain analysis were reported in three-dimensional stereotactic coordinates [Talairach or Montreal Neurological Institute (MNI)]; (6) If the study was a longitudinal or intervention trial, only baseline data were included for analysis.

Exclusion criteria were: (1) patients with EOS were diagnosed with comorbid neurological or other psychiatric disorders; (2) they had fewer than 10 samples in a single group; (3) three-dimensional stereotactic coordinates of the peak of the activation point were unavailable, even after contacting the corresponding author by email or telephone; (4) the baseline data were unavailable; (5) the full-text could not be accessed; (6) If the data of different studies partially or completely overlapped, only the study with larger sample sizes and higher quality were included.

### Data extraction

For each included study, we extracted the following information: (1) peak coordinates and effect values (e.g., *t*-values, etc.) of brain regions significantly different between patients with EOS and HCs; (2) the demographic and clinical characteristics, including sample size, gender, mean age, years of education, diagnostic criteria, medication status, duration of the illness, and PANSS scores; and (3) the imaging characteristics, including MRI scanner, method of analysis, data processing and analysis software used, the full width at half maximum (FWHM) parameter of the smoothing kernel, slice thickness and statistical thresholds used in brain imaging preprocessing.

### Quality assessment

We used a 10-point checklist based on previous meta-analyses to assess the quality of each study included in the current meta-analysis (32–34), which consisted of three modules including demographic and clinical characteristics of patients and HCs, methods of image acquisition and analysis, and quality of results and conclusions. Specifically, scores of 0, 0.5, and 1 were assigned to each item based on whether the criteria were not satisfied, partially satisfied, or fully satisfied, with a total score of no more than 10 (**Supplementary Table S1**). The literature search, study selection, data extraction, and quality assessment were performed independently by two researchers (Lu Wang and Ruishan Liu). Any discrepancies were resolved jointly by the third and fourth researchers (Hongwei Li and Lihua Zhuo) for a final decision.

### Data analyses

#### Voxel-wise meta-analyses for functional and structural differences

We performed separate meta-analyses of brain regions with significant differences in resting-state regional functional brain activity (i.e., ALFF, fALFF, ReHo, and CBF) and structure (i.e., gray matter volume) between patients with EOS and HCs, using the Seed-based *d* Mapping with Permutation of Subject Images (SDM-PSI, version 6.23) (https://www.sdmproject.com/) software following standard procedures. The procedures have been described in detail in previous studies (35, 36). Briefly, we initially created the corresponding text for each study and then entered the peak coordinates and effect sizes of the extracted brain regions that were significantly different between patients with EOS and HCs into the corresponding text files. If the effect sizes reported in the original studies were *p*-values or *Z*-values, we could convert them to *t*-values using the SDM online converter. Meanwhile, if the coordinates reported in Talairach space could be uniformly transformed to MNI space by matrix transformation so that all peak coordinates were in the same normalized space. We then recreated the standardized MNI-based effect size maps (Hedges’ effect size) of contrast results for each dataset separately using an anisotropic non-normalized Gaussian kernel. Next, the mean maps were computed using the random-effects model, weighted by sample size, intra-study variability, and between-study heterogeneity, and multiple imputations were pooled using Rubin’s rules. Finally, the maps were visualized by MRIcron software (www.mricro.com/mricron/). In addition, the meta-analyses were conducted with the default Gaussian kernel size and thresholds of the SDM-PSI software [i.e., FWHM = 20 mm, peak height *Z* > 1, *p* < 0.005 (uncorrected), cluster extent > 10 voxels], which have been validated to balance false positives and false negatives optimally and to be approximate to the corrected results (37–39).

### Multimodal meta-analysis

We overlapped the thresholded meta-analytic results-maps of resting-state regional functional brain activity and brain GMV alterations to localize brain regions presenting abnormalities both at the functional and structural level in patients with EOS (i.e., examine the convergence of the results from different modalities). It is worth noting that in the multimodal analysis, we used a more stringent probability threshold (i.e., *p* < 0.0025) (40).

### Subgroup meta-analyses

In the current study, to verify the reliability of the results of the main meta-analyses, we conducted a validation subgroup analysis after excluding studies with uncorrected statistics. Furthermore, to explore the structural and functional changes in the brain of EOS patients in different statuses, we performed the following two subgroup analyses (1): the studies were conducted in patients with drug-naive EOS; (2) the studies were conducted in patients with first-episode EOS. The same thresholds were applied as for the subgroup meta-analyses [i.e., peak height *Z* > 1, cluster range > 10 voxels, *p* < 0.005 (uncorrected)].

### Analyses of jackknife sensitivity, heterogeneity, and publication bias

In the present study, we used the whole-brain voxel-based Jackknife sensitivity analysis, i.e. iteratively repeating the same analysis after excluding one dataset at a time to evaluate the stability and reproducibility of the main meta-analyses results (41, 42). If an abnormal brain region remains significant in all or most studies, the result is considered highly reproducible and stable. We used the *I^2^* statistic to assess between-study heterogeneity of the results, with *I^2^* < 50% commonly indicating low heterogeneity (43). In addition, to evaluate potential publication bias, in the present study, funnel plots were created for visual inspection, and publication bias was quantified by Egger’s test. Significant publication bias was considered to exist if the *p*-value < 0.05 of Egger’s test and the funnel plots were asymmetric (44, 45).

### Meta-regression analyses

To explore the potential impact of clinical variables (including mean age, percentage of females, years of education, illness duration, and PANSS scores) on the results of the meta-analyses, linear regression analyses were performed in the current study within the EOS. We applied the more conservative thresholds (i.e., *p* < 0.0005 and cluster extent > 10 voxels) recommended by previous studies to minimize the reporting of spurious relationships (46, 47).

## Results

### Included studies and sample characteristics

**Figure 1:**
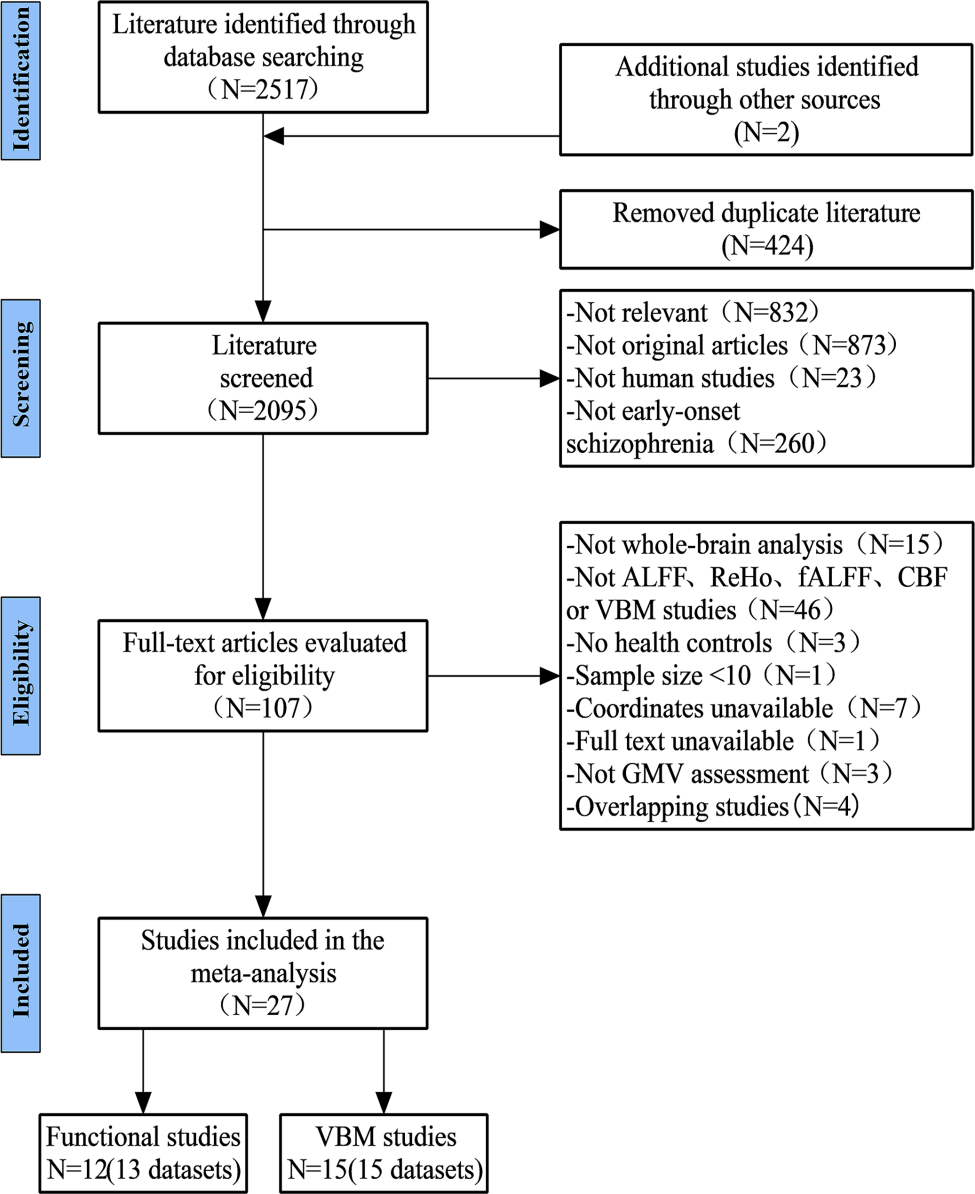
Flow diagram for the identification and exclusion of studies.

The flow chart for identifying and excluding studies is shown in **Figure 1**. Through a systematic and comprehensive search and review, we finally included 12 studies (13 datasets) for resting-state regional functional brain activity and 15 studies (15 datasets) for GMV. The total sample sizes of the functional meta-analysis were 891 subjects, including 496 EOS patients (57.06% females, mean age =14.81 years) and 395 HCs (55.44% females, mean age =14.74 years), and there was no statistically significant difference between the two groups on sex (*χ* ^2^= 0.778, *p*=0.378) or age (*t*=0.172, *p*=0.865). In patients with EOS, the mean duration of illness was 5.74 months (range: 1.50-9.60 months), 86.49% were drug-naive patients, and 83.87% were first-episode patients. In addition, the mean quality score of the included studies was 9.04 (range:7.5-10.0).

The total sample sizes of the structural meta-analysis were 989 subjects, including 458 EOS patients (50.22% females, mean age =16.30 years) and 531 HCs (46.14% females, mean age =16.11 years), and there was no statistically significant difference between the two groups on sex (*χ^2^ =* 1.639, *p*=0.200) or age (*t*=0.177, *p*=0.861). In patients with EOS, the mean duration of illness was 5.76 months (range: 1.20-16.00 months), 50.66% were drug-naive patients, and 77.29% were first-episode patients. Furthermore, the mean quality score of the included studies was 8.80 (range:7.0-9.5). The demographic and clinical characteristics, imaging characteristics, and quality scores of the included studies are presented in **Supplementary Tables S2**, **S3**.

### Main voxel-wise meta-analyses

**Figure 2:**
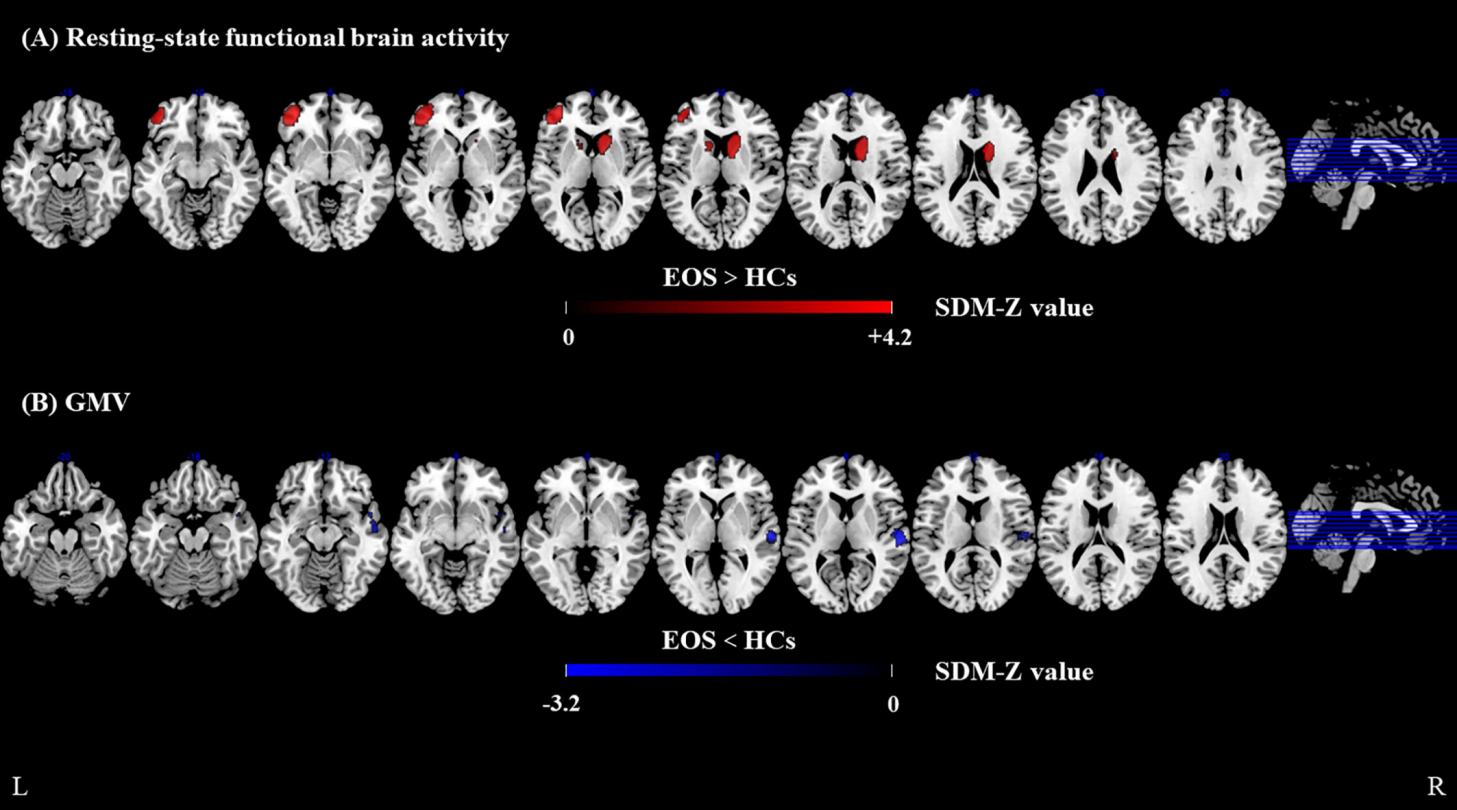
Meta-analyses results of difference between EOS and HCs. **(A)** resting-state regional functional activity difference between EOS and HCs, **(B)** GMV difference between EOS and HCs. Regions with decreased resting-state regional functional activity or GMV are displayed in blue, and regions with increased resting-state regional functional activity or GMV are displayed in red. The color bar indicates the maximum and minimum SDM-Z values. EOS, early-onset schizophrenia; HCs, healthy controls; SDM, Seed-based *d* mapping; GMV, gray matter volume.

In the functional meta-analysis, resting-state functional brain activity was significantly increased in the left middle frontal gyrus (extending to the triangular part of the left inferior frontal gyrus) and the right caudate nucleus in patients with EOS, compared with HCs. However, no brain regions with significantly decreased resting-state brain functional activity were observed (**Figure 2**, **Supplementary Table S4**).

In the structural meta-analysis, GMV was significantly decreased in the right superior temporal gyrus (extending to the right rolandic operculum), the right middle temporal gyrus, and the right temporal pole (superior temporal gyrus) in patients with EOS, compared with HCs. No significantly increased GMV was observed in patients with EOS (**Figure 2**, **Supplementary Table S5**).

### Multimodal meta-analysis

Brain regions with conjoint alterations in resting-state functional brain activity and GMV were not observed in patients with EOS, compared with HCs.

### Subgroup meta-analyses

First, we performed subgroup analyses after excluding studies of uncorrected for statistics. To be specific, for resting-state functional brain activity analysis (10 datasets), compared with HCs, patients with EOS showed significantly increased resting-state functional brain activity in the left middle frontal gyrus (extending to the orbital part of the left inferior frontal gyrus), and right caudate nucleus, while resting-state functional brain activity was significantly decreased in the left superior temporal gyrus (**Supplementary Table S6**, **Supplementary Figure S1**). For GMV analysis (12 datasets), GMV was significantly decreased in the right superior temporal gyrus in patients with EOS, compared with HCs (**Supplementary Table S7**, **Supplementary Figure S1**).

Subgroup analyses of patients with first-episode EOS revealed that compared with HCs, patients with first-episode EOS had significantly increased resting-state regional functional brain activity in the triangular part of the left inferior frontal gyrus (extending to the left middle frontal gyrus) and significantly decreased in the left postcentral gyrus (**Supplementary Table S8**, **Supplementary Figure S2**). However, no brain regions with significantly increased or decreased GMV were observed between patients with first-episode EOS and HCs.

In addition, Subgroup analyses of patients with drug-naive EOS indicated that patients with drug-naive EOS had significantly increased resting-state regional functional brain activity in the left middle frontal gyrus (extending to the triangular part of the left inferior frontal gyrus and the orbital part of the left inferior frontal gyrus) and right caudate nucleus, compared with HCs (**Supplementary Table S9**, **Supplementary Figure S2**). Likewise, no brain regions with significant increased or decreased GMV were observed in patients with drug-naive EOS.

### Analyses of jackknife sensitivity, heterogeneity, and publication bias

The jackknife sensitivity analysis revealed high reliability and reproducibility of the results of the functional and structural meta-analysis. In the functional meta-analysis, jackknife sensitivity analysis showed that alterations (i.e., decreased resting-state functional brain activity) in the left middle frontal gyrus (extending to the triangular part of the inferior frontal gyrus) and the right caudate nucleus remained significant in all combinations. For the structural meta-analysis, jackknife sensitivity analysis indicated that the most robust data was the decreased GMV in the right superior temporal gyrus (extending to the right rolandic operculum), which could be cross-validated in at least 13 of all 15 datasets. In addition, the other results were relatively robust, in which the right middle temporal gyrus could be cross-validated in 8 of the 15 data sets, and the right temporal pole could be cross-validated in 7 of the 15 data sets.

In both functional and structural meta-analyses, no significant between-study heterogeneity (*I^2^* < 50%) or publication bias (Egger’s test, *p* > 0.05) was observed in all brain regions with significant abnormal alterations (**Supplementary Tables S4**, **S5**).

### Meta-regression analyses

Meta-regression analyses demonstrated that there were no significant linear associations between abnormal alterations in resting-state regional functional brain activity or GMV and clinical variables (including age, percentage of females, years of education, illness duration, and disease severity scores) in patients with EOS.

## Discussion

To our knowledge, this is the first multimodal neuroimaging meta-analysis of EOS patients applying the SDM-PSI meta-analysis method. The main findings of the present study are as follows (1): compared with HCs, patients with EOS had significantly increased resting-state regional functional brain activity in the left middle frontal gyrus (extending to the triangular part of the left inferior frontal gyrus) and the right caudate nucleus; (2) the brain regions with significantly decreased GMV in patients with EOS were mainly located in the right superior temporal gyrus (extending to the rolandic operculum), the right middle temporal gyrus, and the right temporal pole (superior temporal gyrus). (3) In the multimodal meta-analysis, we failed to identify brain regions with conjoint abnormalities in resting-state regional functional brain activity and GMV in patients with EOS; (4) The results of jackknife sensitivity analyses, heterogeneity analyses, and validation subgroup analyses showed relatively high reliability and reproducibility of the results of the main meta-analyses; (5) Meta-regression analyses showed no significant linear associations between the major structural and functional alterations in EOS patients and age, gender, years of education, duration of illness, or severity of disease.

In the functional meta-analysis, we identified a significantly increased regional spontaneous brain activity in the left middle frontal gyrus in patients with EOS, which was consistent with the results of the previous studies (48, 49). The middle frontal gyrus is one of the important constituent brain regions of the PFC, which is closely related to working memory (50). Previous studies have shown that working memory processes are often impaired in patients with schizophrenia and their unaffected first-degree relatives (51–53). In addition, abnormal activation patterns in the middle frontal gyrus are frequently associated with attentional control (54), and the top-down attentional control processes of patients with schizophrenia are likewise particularly susceptible to being impaired (55). On the other hand, neural activity in the left middle frontal gyrus was significantly increased in patients with schizophrenia during the processing of fearful faces (56). Athanassiou et al. reported that abnormal activation of the left middle frontal gyrus in suicidal patients with schizophrenia also during emotional processing tasks (57), and significant cortical thinning of the PFC was also observed in suicidal patients with schizophrenia (58), suggesting that suicidal behaviors in patients with schizophrenia may be related to the abnormal anatomical structure and neural activity of the PFC. In summary, the PFC is involved in the regulation of decision-making and executive control, and in appropriate behaviors by integrating feeling and emotional information (59, 60). The hyperactivation of functional activity in the PFC found in the present study we speculate may be due to a functional compensatory neural mechanism that the organism develops after cognitive, emotional processing and behavioral processes are impaired in patients with EOS, and genetic factors may also influence some of these processes. However, it is noteworthy that some studies reported significantly decreased regional spontaneous functional activity in the PFC in patients with EOS (23, 61). The possible reasons for this discrepancy are: (i) the irregular involvement of the PFC may not be fully explained by an explicit failure of function in this region, but perhaps reflects a dysfunction in the processes of cognitive control, affective processing, and behavioral regulation themselves; (ii) heterogeneity of the samples, moderating variables, and performance on the task (62).

The triangular part of the inferior frontal gyrus belongs to Broca’s area, the area where the motor speech center is located and is closely related to the processing of verbal information and the production of discourse (63, 64). Patients with schizophrenia often suffer from extensive language disorders (65). Previous studies revealed that abnormal activation of the left inferior frontal gyrus was negatively correlated with increased use of transitive verbs in continuous speech in patients with schizophrenia (66). It was also found that patients with schizophrenia had hyperactivation of the left inferior frontal gyrus during a speech task (67). In addition, a significantly increased spontaneous neural activity in the triangular part of the left inferior frontal gyrus was positively correlated with the polygenic risk score (PRS) in patients with non-chronic schizophrenia, and furthermore, glutamatergic-related genes involved in synaptic organization and transmission were found to be highly enriched in the PRS-schizophrenia genes (68). A previous study revealed that glutamatergic neurotransmission may play an important role in regulating speech processing and production (69). On the other hand, the left inferior frontal gyrus of young people at family high risk (FHR) for schizophrenia also showed hyperenhancement due to semantic associations (70). Moreover, significantly increased ReHo in the left inferior frontal gyrus showed high sensitivity and specificity in distinguishing treatment-resistant schizophrenia (TRS) from non-treatment-resistant schizophrenia (NTRS) (71). Previous studies also revealed that a significantly increased functional activity in the triangular part of the left inferior frontal gyrus also played an important role in auditory verbal hallucinations (AVH) in the schizophrenia (72). Besides that, a meta-analysis also showed that a significant decreased in GMV in the left inferior frontal gyrus was associated with the development of the AVH (73). In the present study, we identified a significantly increased regional spontaneous neural activity in the triangular part of the left inferior frontal gyrus in patients with EOS, which is consistent with previous studies, suggesting that abnormal neural activity in this region may be involved in the pathogenesis of EOS, which may lead to language deficits and hallucinations and may be a potential biomarker for distinguishing among TRS, NTRS, and HCs and for predicting neurobiological risk of the schizophrenia.

In addition, we also identified the presence of abnormally increased spontaneous neural activity in the right caudate nucleus in patients with EOS, which was consistent with previous findings in patients with adult schizophrenia (74, 75). The caudate nucleus is an important component of the striatum, the vast majority of which is involved in constituting the dorsal striatum, while its ventral portion is one of the components of the ventral striatum. Several studies suggested that striatum dysfunction played a central role in the pathophysiological mechanisms of schizophrenia (76–78). The striatum mainly received dopaminergic neural projections from the midbrain (especially the substantia nigra and ventral tegmental area (79). Previous studies showed that striatal dopamine synthesis and release were significantly enhanced in patients with schizophrenia and were associated with positive symptoms of schizophrenia (80, 81). And the increased volume of the striatum may be the structural basis for the hyperfunction of the striatal dopaminergic system in the schizophrenia (82). Sorg et al. revealed that increased intrinsic neural activity in the striatum of patients with schizophrenia corresponded to symptom dimensions and disorder states, and further identified a potential link between intrinsic neural activity in the striatum and signaling in the dopamine pathway (83). An animal study also found that intrinsic neural activity in the striatum was modulated by dopamine levels (84). In addition, abnormalities of spontaneous functional activity in the striatum may cause dysfunction of the frontal cortex-thalamus-striatum-midbrain circuit, leading to imbalances in the signaling of neurotransmitters, such as dopamine, which can reduce the signal-to-noise ratio of neural activity and impair cortical and basal ganglia function resulting in the development of psychotic symptoms (85, 86). However, it is worth noting that direct evidence of the association of intrinsic neural activity in the striatum with dopamine signaling is still lacking, which awaits further studies in the future. Our findings similarly indicated that striatal dysfunction may play a key role in the pathogenesis of schizophrenia and provided evidence for the EOS aspect of the schizophrenia dopamine hypothesis.

In the structural meta-analysis, the present study revealed that compared with HCs, the regions with significantly decreased GMV in patients with EOS were mainly located in the right superior temporal gyrus (extending to the right rolandic operculum), the right middle temporal gyrus, and the right temporal pole (superior temporal gyrus). superior temporal gyrus plays a crucial role in auditory information processing, language processing and auditory memory (87). Several previous studies showed a strong association between GMV changes in superior temporal gyrus and auditory verbal hallucinations (AVH) (88–90). To be specific, Zhang et al. reported a negative correlation between decreased GMV in superior temporal gyrus and the severity of AVH symptoms (91). Some studies also revealed functional sub-regions of the superior temporal gyrus, e.g. Heschl’s gyrus (primary auditory cortex) and planum temporale were similarly altered, and further found that both were associated with AVH and delusional behaviors in the schizophrenia (92–94). In addition, in the present study, we also identified that GMV abnormalities in the right superior temporal gyrus extended to the right rolandic operculum. The rolandic operculum is located in the frontal lobe and also plays a role in auditory feedback processing, suggesting that decreased GMV in the right rolandic operculum may be associated with abnormal auditory feedback processing behind AVH (95). On the other hand, previous VBM studies in adult schizophrenia patients with first-episode and antipsychotic-naive also revealed significantly decreased GMV in the right superior temporal gyrus (96, 97), suggesting that GMV alterations in the right superior temporal gyrus may be a stable biomarker for schizophrenia less susceptible to time of onset. A structural meta-analysis showed that a significantly decreased GMV in the right superior temporal gyrus was similarly found in a population at high risk of clinical psychosis (and later converted to psychosis) (98). Moreover, decreased GMV in superior temporal gyrus was strongly associated with the severity of negative and psychotic symptoms in schizophrenia patients (99). However, unfortunately, the present study didn’t find a significant association (at least not linearly) between GMV alterations and schizophrenia symptoms severity, which may be explained by the fact that most of the patients with EOS included in the present study were first-episode patients (with a relatively short duration of illness), the relatively small sample size or the inherent limitations of cross-sectional studies as opposed to longitudinal studies (i.e., additional timepoints per subject may strengthen the observed associations) (100). Previous studies also identified a positive correlation between GMV alterations in the right superior temporal gyrus and the PANSS total scores reduction ratio in patients with schizophrenia after treatment with antipsychotic medication (101, 102), suggesting that GMV alterations in the right superior temporal gyrus may be a potential biomarker for predicting the efficacy of schizophrenia drug therapy. Furthermore, significant abnormalities in GMV of the right temporal pole (superior temporal gyrus) were found in the present study. The temporal pole is mainly involved in the integration of facial information processing and emotional information processing (103). Previous studies indicated lower GMV in the temporal pole region in schizophrenia patients with a history of violent behaviors compared to schizophrenia patients without a history of violent behaviors (104). Therefore, we hypothesized that decreased GMV in the temporal pole in schizophrenia may contribute to a higher risk of violence by disturbing the process of integration of facial and emotional information processing.

The middle temporal gyrus is also one of the vulnerable brain regions in patients with schizophrenia (105), and the middle temporal gyrus plays a key role in language comprehension, semantic reasoning, and integrating information from different semantic systems (106, 107). Thus, patients with schizophrenia often have extensive social cognitive deficits, especially in interpersonal communication (108). In this study, we found that patients with EOS also presented with a GMV deficit in the middle temporal gyrus, which was similar to the results of previous studies (109, 110). Moreover, it was also found that both patients with schizophrenia and their first-degree unaffected siblings had significantly decreased regional GMV in the left middle temporal gyrus relative to HCs and that the mean GMV in the left middle temporal gyrus was a good predictor in distinguishing patients/siblings from HCs (111). Another study identified a correlation between genetically determined IL-6 levels and decreased GMV in the middle temporal gyrus (112). Furthermore, the association of decreased GMV in the right middle temporal gyrus with the Val158Met polymorphism in the COMT gene was also reported (113, 114). The above-mentioned studies suggested that decreased GMV in the middle temporal gyrus may be a potential endophenotype of schizophrenia with some heritability and specificity. In addition, decreased GMV in the middle temporal gyrus was associated with a poor prognosis in the schizophrenia (115).

## Limitation

The present study had some limitations. First, the present meta-analysis was based on the peak coordinates and effect sizes of significantly abnormal brain regions reported in the original studies rather than the original statistical maps, which resulted in the loss of some information and may have decreased the accuracy of the results of the present study to some extent. Second, the majority of subjects included in the present study were from the Chinese population, which somewhat limited the generalizability of the results to other populations. Third, because the vast majority of the included studies were cross-sectional and we included only baseline data for longitudinal studies, we were not able to determine whether alterations in brain anatomical structure and function were part of the pathogenesis of EOS or a consequence of the disease. Fourth, since the original studies included in the present study were limited and didn’t meet the criteria for subgroup analysis, we didn’t perform a subgroup analysis of the different functional imaging methods in the rs-fMRI studies, and more high-quality studies will need to be included in the future to explore the impact of functional imaging methods on the results of the main meta-analyses. Furthermore, we conducted separate subgroup analyses of first-episode EOS patients and drug-naive EOS patients which indicated that alterations of resting-state regional functional brain activity in patients with EOS seemed to be related not to the time of onset, or the medication status, but to the disease itself. Finally, Finally, in the subgroup analyses, some of the results were inconsistent with those of the main meta-analyses, and we failed to identify brain regions with significantly increased or decreased regional GMV either in the subgroup analysis of patients with first-episode EOS or in the subgroup analysis of patients with drug-naive EOS, possibly due to the presence of some heterogeneities that were not captured by the present meta-analysis (e.g., type of antipsychotic medication used, etc.), the limited inclusion of the sample sizes, the between-subjects heterogeneity, and methodological heterogeneity among the included studies (e.g., scanning methods and parameters, methods of data processing and analysis, etc.). Hence, in the future, studies with larger and more homogeneous samples are needed to further validate the findings of the present study.

## Conclusion

In summary, EOS showed significant abnormalities in resting-state functional brain activity in the left middle frontal gyrus (extending to the triangular part of the left inferior frontal gyrus) and the right caudate nucleus, and significant gray matter structural deficits in the right superior temporal gyrus (extending to the right rolandic operculum), the right middle temporal gyrus, and the right temporal pole (superior temporal gyrus). These findings may contribute to our more comprehensive understanding of the underlying pathophysiological mechanisms of schizophrenia and provide potential biomarkers for the diagnosis, differential diagnosis and treatment of schizophrenia.

## Data Availability

The original contributions presented in the study are included in the article/**Supplementary Material**. Further inquiries can be directed to the corresponding authors.
