# Supplementary material for: Meta-analysis of structural and functional brain abnormalities in early-onset schizophrenia
Source: Front Psychiatry. 2024 Aug 23;15:1465758. doi: 10.3389/fpsyt.2024.1465758 (PMC11377232; doi:10.3389/fpsyt.2024.1465758)
Supplement: Supplementary file 1 [file DataSheet1.docx]

Supplementary Material

**Table S1 Quality assessment checklist (score 0/0.5/1 per item; total score out of 10)***

| Category 1: Participants |
| --- |
| 1. Patients were evaluated prospectively, specific diagnostic criteria were applied, and demographic data were reported. |
| 2. Healthy comparison participants were evaluated prospectively, psychiatric and medical illnesses were excluded. |
| 3. Important variables (e.g., age, sex, illness duration, onset, medication status, comorbidity, severity of illness) were checked either by stratification or statistically. |
| 4. Sample size per group > 10. |
| Category 2: Methods for image acquisition and analysis |
| 5. Whole brain analysis was automated with no a priori regional selection. |
| 6. Coordinates reported in a standard space. |
| 7. The imaging technique used was clearly described so that it could be reproduced. |
| 8. Measurements were clearly described so that they could be reproduced. |
| Category 3: Results and conclusions |
| 9. Statistical parameters for significant and important nonsignificant differences were provided. |
| 10. Conclusions were consistent with the results obtained and the limitations were discussed. |
| *When criteria were partially met, 0.5 points were awarded. |

**Table S2 Demographic and clinical characteristics of included studies.**

| **Study** | | **Subjects**  **(female)** | | | |  | | **Mean age/y** | | | |  | | **Education/y** | | | | | **Diagnosis criteria** | | **Illness duration/m** | | **PANSS**  **scores** | | | | | | **Medication (%)** | | **First -episode** | |  |
| --- | --- | --- | --- | --- | --- | --- | --- | --- | --- | --- | --- | --- | --- | --- | --- | --- | --- | --- | --- | --- | --- | --- | --- | --- | --- | --- | --- | --- | --- | --- | --- | --- | --- |
|  |  | **EOS** | | **HCs** | |  | | **EOS** | | **HCs** | |  | | **EOS** | | | **HCs** | |  | |  | | **Total** | | **Positive** | | **Negative** | |  | |  | |  |
| **Resting-state functional brain activity studies** | | | | | | | | | | | | | | | | | | | | | | | | | | | | | | | | |  |
| Jiang  et al^(1)^. (2010) | | | 18(9) | | 18(9) | |  | | 16.30 | | 16.40 | |  | | 9.60 | | 9.50 | | | DSM-IV | | 9.60 | | 91.80 | | NA | | NA | | 78 | | YES | |
|  | | | | | | | | | | | | | | | | | | | | | | | | | | | | | | | | | |
| Zheng  et al^(2)^. (2016) | | 35(15) | | 30(17) | |  | | 15.50 | | 15.43 | |  | | 8.70 | | 8.50 | | | DSM-IV | | 6.60 | | 74.62 | | 20.42 | | 20.91 | | Drug-naive | | YES | |  |
|  | | | | | | | | | | | | | | | | | | | | | | | | | | | | | | | | |  |
| Xiong  et al^(3)^. (2016) | | 20(12) | | 20(11) | |  | | 14.30 | | 14.40 | |  | | 7.30 | | 7.40 | | | NA | | 8.90 | | 71.30 | | NA | | NA | | Drug-naive | | YES | |  |
|  | | | | | | | | | | | | | | | | | | | | | | | | | | | | | | | | |  |
| Lü  et al^(4).^ (2016) | | 50(27) | | 33(18) | |  | | 14.20 | | 14.30 | |  | | 8.20 | | 8.30 | | | DSM-IV | | 5.30 | | NA | | NA | | NA | | Drug-naive | | YES | |  |
|  | | | | | | | | | | | | | | | | | | | | | | | | | | | | | | | | |  |
| Liang  et al^(5)^. (2018) | | 30(19) | | 30(16) | |  | | 13.00 | | 12.90 | |  | | NA | | NA | | | DSM-IV | | 5.50 | | 74.50 | | NA | | NA | | Drug-naive | | NA | |  |
|  | | | | | | | | | | | | | | | | | | | | | | | | | | | | | | | | |  |
| Pei  et al^(6)^. (2018) | | 33(16) | | 34(17) | |  | | 15.30 | | 15.60 | |  | | NA | | NA | | | DSM-IV | | NA | | NA | | NA | | NA | | Drug-naive | | YES | |  |
|  | | | | | | | | | | | | | | | | | | | | | | | | | | | | | | | | |  |
| Wang  et al^(7)^. (2018) | | 48(27) | | 31(17) | |  | | 15.79 | | 15.42 | |  | | 8.88 | | 8.44 | | | DSM-IV | | 5..35 | | 75.10 | | 21.50 | | 17.92 | | Drug-naive | | YES | |  |
|  | | | | | | | | | | | | | | | | | | | | | | | | | | | | | | | | |  |
| Li^a^  et al^(8)^. (2019) | | 26(21) | | 33(19) | |  | | 14.00 | | 13.58 | |  | | 6.46 | | 6.12 | | | DSM-IV | | 2.00 | | 89.77 | | 26.42 | | 24.58 | | Drug-naive | | YES | |  |
|  | | | | | | | | | | | | | | | | | | | | | | | | | | | | | | | | |  |
| Li^a^  et al^(8)^. (2019) | | 26(16) | | 33(19) | |  | | 13.96 | | 13.58 | |  | | 6.38 | | 6.12 | | | DSM-IV | | 1.50 | | 93.65 | | 23.27 | | 24.81 | | Drug-naive | | YES | |  |
|  | | | | | | | | | | | | | | | | | | | | | | | | | | | | | | | | |  |
| Lyu  et al^(9)^. (2021) | | 32(17) | | 27(17) | |  | | 16.75 | | 16.40 | |  | | 10.19 | | 10.11 | | | DSM-IV | | 9.19 | | 79.44 | | 22.75 | | 16.97 | | Drug-naive | | YES | |  |
|  | | | | | | | | | | | | | | | | | | | | | | | | | | | | | | | | |  |
| Li  et al^(10)^. (2021) | | 79(51) | | 32(18) | |  | | 14.10 | | 13.70 | |  | | 6.60 | | 6.80 | | | DSM-IV | | 3.90 | | 90.20 | | 24.40 | | 22.90 | | Drug-naive | | YES | |  |
|  | | | | | | | | | | | | | | | | | | | | | | | | | | | | | | | | |  |
| Yang  et al^(11)^. (2022) | | 49(30) | | 41(26) | |  | | 15.16 | | 15.57 | |  | | NA | | NA | | | DSM-IV | | NA | | 87.15 | | 23.36 | | 22.90 | | NA | | YES | |  |
|  | | | | | | | | | | | | | | | | | | | | | | | | | | | | | | | | |  |
| Liang  et al^(12)^. (2024) | | 50(23) | | 33(15) | |  | | 14.22 | | 14.33 | |  | | 8.22 | | 8.33 | | | DSM-IV | | 5.26 | | NA | | NA | | NA | | Drug-naive | | NA | |  |
| **GMV studies** | | | | | | | | | | | | | | | | | | | | | | | | | | | | | | | | |  |
| Douaud  et al^(13)^. (2007) | | 25(7) | | 25(8) | |  | | 16.33 | | 16.01 | |  | | NA | | | NA | | DSM-IV | | 1.40 | | NA | | 22.00 | | 16.00 | | 100 | | NA | |  |
|  | | | | | | | | | | | | | | | | | | | | | | | | | | | | | | | | |  |
| Pagsberg  et al^(14)^. (2007) | | 15(8) | | 29(18) | |  | | 15.60 | | 16.00 | |  | | NA | | | NA | | ICD-10 | | NA | | NA | | NA | | NA | | NA | | YES | |  |
|  | | | | | | | | | | | | | | | | | | | | | | | | | | | | | | | | |  |
| Yoshihara  et al^(15)^. (2008) | | 18(9) | | 18(9) | |  | | 15.80 | | 15.80 | |  | | NA | | | NA | | DSM-IV | | 1.20 | | NA | | 13.80 | | 19.10 | | 94 | | NA | |  |
|  | | | | | | | | | | | | | | | | | | | | | | | | | | | | | | | | |  |
| Janssen  et al^(16)^. (2008) | | 25(6) | | 51(16) | |  | | 15.40 | | 15.40 | |  | | 8.00 | | | 8.70 | | DSM-IV | | NA | | 99.50 | | 25.70 | | 25.60 | | NA | | YES | |  |
|  | | | | | | | | | | | | | | | | | | | | | | | | | | | | | | | | |  |
| Wei  et al^(17)^. (2011) | | 21(8) | | 22(9) | |  | | 20.30 | | 20.20 | |  | | 10.00 | | | 10.20 | | CCMD | | NA | | 71.90 | | NA | | NA | | Drug-naive | | NA | |  |
|  | | | | | | | | | | | | | | | | | | | | | | | | | | | | | | | | |  |
| Tang  et al^(18)^. (2012) | | 29(16) | | 34(18) | |  | | 16.50 | | 16.60 | |  | | 9.70 | | | 9.70 | | DSM-IV | | 9.30 | | 77.70 | | 22.30 | | 20.80 | | 79 | | YES | |  |
|  | | | | | | | | | | | | | | | | | | | | | | | | | | | | | | | | |  |
| Fang  et al^(19)^. (2015) | | 20(8) | | 20(8) | |  | | 24.65 | | 24.95 | |  | | 12.15 | | | 13.50 | | CCMD | | NA | | NA | | NA | | NA | | Drug-naive | | NA | |  |
|  | | | | | | | | | | | | | | | | | | | | | | | | | | | | | | | | |  |
| Zhang  et al^(20)^. (2015) | | 37(20) | | 30(13) | |  | | 15.50 | | 15.30 | |  | | 8.50 | | | 8.70 | | DSM-IV | | 16.00 | | 74.62 | | 20.42 | | 20.91 | | Drug-naive | | YES | |  |
|  | | | | | | | | | | | | | | | | | | | | | | | | | | | | | | | | |  |
| Zhang  et al^(21)^. (2017) | | 26(13) | | 26(13) | |  | | 16.87 | | 16.81 | |  | | 10.35 | | | 11.19 | | DSM-IV | | 3.61 | | 93.42 | | 25.12 | | 20.46 | | Drug-naive | | YES | |  |
|  | | | | | | | | | | | | | | | | | | | | | | | | | | | | | | | | |  |
| Zhang  et al^(22)^. (2018) | | 34(18) | | 32(16) | |  | | 15.70 | | 15.40 | |  | | 9.30 | | | 8.90 | | DSM-IV | | NA | | 88.50 | | 26.00 | | 20.10 | | Drug-naive | | YES | |  |
|  | | | | | | | | | | | | | | | | | | | | | | | | | | | | | | | | |  |
| Castro‑Fornieles  et al^(23)^. (2018) | | 34(10) | | 70(22) | |  | | 15.20 | | 15.30 | |  | | 8.20 | | | 9.00 | | NA | | 4.10 | | 89.20 | | 24.50 | | 20.60 | | NA | | YES | |  |
|  | | | | | | | | | | | | | | | | | | | | | | | | | | | | | | | | |  |
| Gao  et al^(24)^. (2019) | | 39(22) | | 30(16) | |  | | 13.50 | | 13.30 | |  | | 6.30 | | | 6.30 | | DSM-IV | | 5.00 | | NA | | NA | | NA | | Drug-naive | | YES | |  |
|  | | | | | | | | | | | | | | | | | | | | | | | | | | | | | | | | |  |
| Shen  et al^(25)^. (2021) | | 20(11) | | 25(13) | |  | | 13.95 | | 12.68 | |  | | NA | | | NA | | DSM-V | | NA | | 77.07 | | 17.07 | | 21.07 | | NA | | NA | |  |
|  | | | | | | | | | | | | | | | | | | | | | | | | | | | | | | | | |  |
| Li  et al^(26)^. (2022) | | 55(35) | | 79(46) | |  | | 14.90 | | 14.30 | |  | | NA | | | NA | | DSM-IV | | NA | | 65.40 | | 14.90 | | 13.90 | | Drug-naive | | YES | |  |
|  | | | | | | | | | | | | | | | | | | | | | | | | | | | | | | | | |  |
| Cai  et al^(27)^. (2022) | | 60(39) | | 40(20) | |  | | 14.27 | | 13.60 | |  | | 8.13 | | | 7.68 | | DSM-IV | | 5.43 | | 79.18 | | 21.43 | | 19.92 | | 65 | | YES | |  |

*Note:* ^a^The study included two subgroups, which we treated as two separate datasets.

*Abbreviation*: EOS, early-onset schizophrenia; HCs, healthy controls; y, year; m, month; NA, not available; PANSS, Positive and Negative Syndrome Scale; DSM, Diagnostic and Statistical Manual of Mental Disorders; ICD, International Statistical Classification; CCMD, Chinese classification of mental disorders.

**Table S3 Imaging characteristics and quality scores of included studies.**

| **Study** | **MRI scanner** | **Method of analysis** | | **Software** | | **Slice thickness**  **(mm)** | | **FWHM (mm)** | | **Statistical threshold** | | **Quality scores** | |  |
| --- | --- | --- | --- | --- | --- | --- | --- | --- | --- | --- | --- | --- | --- | --- |
| **Resting-state functional brain activity studies** | | | | | | | | | | | | | |  |
| Jiang  et al^(1)^. (2010) | 1.5T | | ReHo | | SPM5  REST | | 5 | | 4 | | FDR(*p*_corrected_＜0.05) | | 8.5 | |
|  | | | | | | | | | | | | | | |
| Zheng  et al^(2)^. (2016) | 3.0T | | ALFF | | DPARSF  SPM8  REST | | NA | | 6 | | AlphaSim(*p*_corrected_＜0.05) | | 9.5 | |
|  | | | | | | | | | | | | | | |
| Xiong  et al^(3)^. (2016) | 3.0T | | ALFF | | DPARSF  SPM8  REST | | 4 | | NA | | AlphaSim(*p*_corrected_＜0.05) | | 9.0 | |
|  | | | | | | | | | | | | | | |
| Lü  et al^(4)^. (2016) | 3.0T | | fALFF | | DPARSF SPM8  REST | | 4 | | 6 | | AlphaSim(*p*_corrected_＜0.05) | | 8.5 | |
|  | | | | | | | | | | | | | | |
| Liang  et al^(5)^. (2018) | 3.0T | | ALFF | | REST | | 1 | | NA | | AlphaSim(*p*_corrected_＜0.05) | | 9.0 | |
|  | | | | | | | | | | | | | | |
| Pei  et al^(6)^. (2018) | 3.0T | | ALFF | | REST | | 4 | | NA | | *p*_NA_＜0.01 | | 7.5 | |
|  | | | | | | | | | | | | | | |
| Wang  et al^(7)^. (2018) | 3.0T | | ReHo | | REST  DPARSF | | 4 | | 4 | | GRF(*p*_corrected_＜0.005) | | 10.0 | |
|  | | | | | | | | | | | | | | |
| Li^a^  et al^(8)^. (2019) | 3.0T | | ALFF | | DPARSF  SPM  REST | | NA | | 6 | | AlphaSim(*p*_corrected_＜0.05) | | 9.0 | |
|  | | | | | | | | | | | | | | |
| Li^a^  et al^(8)^. (2019) | 3.0T | | ALFF | | DPARSF  SPM  REST | | NA | | 6 | | AlphaSim(*p*_corrected_＜0.05) | | 9.0 | |
|  | | | | | | | | | | | | | | |
| Lyu  et al^(9)^. (2021) | 3.0T | | ReHo | | DPARSF  SPM12  REST | | 1 | | 6 | | AlphaSim(*p*_corrected_＜0.05) | | 9.5 | |
|  | | | | | | | | | | | | | | |
| Li  et al^(10)^. (2021) | 3.0T | | ReHo | | DPARSF  REST | | 4 | | 6 | | *p*_NA_＜0.05 | | 9.0 | |
|  | | | | | | | | | | | | | | |
| Yang  et al^(11)^. (2022) | 3.0T | | ReHo | | SPM | | 4 | | 6 | | AlphaSim(*p*_corrected_＜0.05) | | 9.5 | |
|  |  | |  | |  | |  | |  | |  | |  | |
| Liang  et al^(12)^. (2024) | 3.0T | | ALFF | | SPM8  REST | | 1 | | NA | | AlphaSim(*p*_corrected_＜0.05) | | 9.5 | |
| **GMV studies** | | | | | | | | | | | | | | |
| Douaud  et al^(13)^. (2007) | 1.5T | | VBM | | FSL | | NA | | 1 | | NA(*p*_corrected_＜0.01) | | 7.5 | |
|  | | | | | | | | | | | | | | |
| Pagsberg  et al^(14)^. (2007) | 1.5T | | VBM | | SPM99 | | 12 | | NA | | NA(*p*_corrected_＜0.05) | | 9.0 | |
|  | | | | | | | | | | | | | | |
| Yoshihara  et al^(15)^. (2008) | 1.5T | | VBM | | BAAM | | 2 | | 3 | | FWE(*p*_corrected_=0.001) | | 9.0 | |
|  | | | | | | | | | | | | | | |
|  | | | | | | | | | | | | | | |
| Janssen  et al^(16)^. (2008) | 1.5T | | VBM | | SPM2 | | 12 | | 1.5 | | NA(*p*_corrected_＜0.05) | | 9.0 | |
|  | | | | | | | | | | | | | | |
| Wei  et al^(17)^. (2011) | 1.5T | | VBM | | SPM5 | | 12 | | 1.8 | | *P*_uncorrected_＜0.001 | | 8.5 | |
|  | | | | | | | | | | | | | | |
| Tang  et al^(18)^. (2012) | 1.5T | | VBM | | SPM5 | | 8 | | 1.8 | | FDR(*p*_corrected_＜0.05) | | 9.0 | |
|  | | | | | | | | | | | | | | |
| Fang  et al^(19)^. (2015) | 3.0T | | VBM | | SPM8 | | 8 | | 1 | | FDR(*p*_corrected_＜0.05) | | 7.0 | |
|  | | | | | | | | | | | | | | |
| Zhang  et al^(20)^. (2015) | 3.0T | | VBM | | SPM8 | | 8 | | NA | | AlphaSim(*p*_corrected_＜0.05) | | 9.5 | |
|  | | | | | | | | | | | | | | |
| Zhang  et al^(21)^. (2017) | 3.0T | | VBM | | SPM8 | | 4 | | 1 | | FWE(*p*_corrected_＜0.05) | | 9.0 | |
|  | | | | | | | | | | | | | | |
| Zhang  et al^(22)^. (2018) | 3.0T | | VBM | | SPM8 | | 8 | | 1 | | *p*_uncorrected_＜0.001 | | 9.0 | |
|  | | | | | | | | | | | | | | |
| Castro‑Fornieles  et al^(23)^. (2018) | NA | | VBM | | SPM8 | | NA | | NA | | FWE(*p*_corrected_＜0.05) | | 9.0 | |
|  | | | | | | | | | | | | | | |
| Gao  et al^(24)^. (2019) | 3.0T | | VBM | | SPM8  REST | | 6 | | 1 | | *p*_NA_＜0.05 | | 8.5 | |
|  | | | | | | | | | | | | | | |
| Shen  et al^(25)^. (2021) | 3.0T | | VBM | | CAT12 | | 6 | | 1 | | FWE(*p*_corrected_＜0.05) | | 9.0 | |
|  | | | | | | | | | | | | | | |
| Li  et al^(26)^. (2022) | 3.0T | | VBM | | SPM12 | | 6 | | 3 | | FDR(*p*_corrected_＜0.05) | | 9.5 | |
|  | | | | | | | | | | | | | | |
| Cai  et al^(27)^. (2022) | 3.0T | | VBM | | SPM12 | | 8 | | 1 | | FDR(*p*_corrected_＜0.05) | | 9.5 | |

*Note:* ^a^The study included two subgroups, which we treated as two separate datasets.

*Abbreviation*: MRI, magnetic resonance imaging; FWHM, full width at half maximum; T, Tesla; ReHo, regional homogeneity; ALFF, amplitude of low-frequency fluctuations; fALFF, fractional amplitude of low-frequency fluctuations; NA, not available; DPARSF, data processing assistant for resting-state fMRI; REST, resting-state fMRI data analysis toolkit; SPM, statistical parametric mapping; FDR, false discovery rate; GRF, Gaussian random fields; VBM, voxel-based morphometry; FSL, FMRIB’s Software Library, the University of Oxford; CAT, Computational Anatomy Toolbox; BAAM, Brain Analysis Morphological Mapping; NA, not available; FWE, family wise error.

**Table S4 Resting-state functional brain activity differences between EOS patients compared to HCs.**

| **Local Maximum** |  |  |  |  | **Cluster** | | **Egger’s test (*p* value)** | **Heterogeneity (*I*^2^)** |
| --- | --- | --- | --- | --- | --- | --- | --- | --- |
| **Region** | **Peak MNI coordinate**  **(x, y, z)** | **SDM-Z**  **value** | ***p* value** |  | **No. of voxels** | **Breakdown (No. of voxels)** |  |  |
| ***EOS vs. HCs (EOS＞HCs)*** | | | | | | | | |
| Right caudate nucleus | 12, 4, 18 | 4.15 | 1.70e-5 |  | 612 | Right anterior thalamic projections (290)  Right caudate nucleus (199)  Right caudate nucleus, BA 25 (43)  Corpus callosum (33)  (undefined) (47) | 0.81 | 28.22% |
|  |  |  |  |  |  |  |  |  |
| Left middle frontal gyrus, BA 10 | -38, 50, 4 | 4.01 | 2.98e-5 |  | 754 | Left inferior frontal gyrus, triangular part, BA 45 (149)  Left anterior thalamic projections (102)  Left middle frontal gyrus, BA 46 (100)  Left inferior frontal gyrus, orbital part, BA 47 (79)  Left middle frontal gyrus, orbital part, BA 46 (79)  Left middle frontal gyrus, orbital part, BA 47 (64)  Left middle frontal gyrus, BA 10 (50)  Left inferior network, inferior fronto-occipital fasciculus (38)  Left inferior frontal gyrus, orbital part, BA 46 (19)  Left inferior frontal gyrus, triangular part, BA 46 (18)  Left middle frontal gyrus, BA 47 (16)  Left inferior frontal gyrus, triangular part, BA 47 (9)  Left middle frontal gyrus, BA 45 (7)  Left inferior frontal gyrus, orbital part, BA 45 (7)  Left middle frontal gyrus, orbital part, BA 10 (5)  Left middle frontal gyrus, orbital part 3  Left superior frontal gyrus, dorsolateral, BA 10 (2)  Left superior frontal gyrus, dorsolateral, BA 11 (1)  Left middle frontal gyrus, orbital part, BA 45 (1)  Left middle frontal gyrus, BA 11 (1)  Left inferior frontal gyrus, triangular part (1)  Corpus callosum (1)  Left superior frontal gyrus, orbital part, BA 47 (1)  (undefined) (1) | 0.73 | 8.71% |
| Left anterior thalamic projections | -16, 12, 8 | 2.88 | 6.14e-4 |  | 49 | Left anterior thalamic projections (39)  Left caudate nucleus (9)  Left caudate nucleus, BA 25 (1) | 0.79 | 20.93% |

**Abbreviations:** HCs, healthy controls; EOS, early-onset schizophrenia; MNI, Montreal Neurological Institute; SDM, seed-based *d* mapping; BA, Brodmann area.

**Table S5 GMV differences between EOS patients compared to HCs.**

| **Local Maximum** |  |  |  |  | **Cluster** | | **Egger’s test (*p* value)** | **Heterogeneity (*I*^2^)** |
| --- | --- | --- | --- | --- | --- | --- | --- | --- |
| **Region** | **Peak MNI coordinate**  **(x, y, z)** | **SDM-Z**  **value** | ***p* value** |  | **No. of voxels** | **Breakdown (No. of voxels)** |  |  |
| ***EOS vs. HCs (EOS＜HCs)*** | | | | | | | | |
| Right superior temporal gyrus, BA 22 | 58, -22, 8 | -3.17 | 7.58e-4 |  | 177 | Right superior temporal gyrus, BA 22 (54)  Corpus callosum (50)  Right superior temporal gyrus, BA 48 (34)  Right rolandic operculum, BA 48 (21)  Right heschl gyrus, BA 48 (7)  Right superior temporal gyrus (7)  Right rolandic operculum (3)  Right superior temporal gyrus, BA 42 (1) | 0.63 | 9.08% |
| Right middle temporal gyrus, BA 22 | 58, -10, -12 | -2.89 | 1.91e-3 |  | 37 | Right superior temporal gyrus, BA 22 (23)  Right middle temporal gyrus, BA 22 (7)  Right superior temporal gyrus, BA 21 (5)  Corpus callosum (1)  Right middle temporal gyrus, BA 21 (1) | 0.98 | 1.66% |
| Right temporal pole, superior temporal gyrus, BA 21 | 50, 6, -14 | -2.77 | 2.79e-3 |  | 23 | Right temporal pole, superior temporal gyrus, BA 38 (9)  Right temporal pole, superior temporal gyrus, BA 21 (4)  Right insula, BA 48 (3)  Right temporal pole, superior temporal gyrus (3)  Right insula (2)  (undefined) (2) | 0.67 | 0.97% |

**Abbreviations:** HCs, healthy controls; EOS, early-onset schizophrenia; MNI, Montreal Neurological Institute; SDM, seed-based *d* mapping; BA, Brodmann area.

**Table S6 Resting-state functional brain activity differences between EOS patients compared to HCs (excluding studies of uncorrected for statistics).**

| **Local Maximum** |  |  |  |  | **Cluster** | | |
| --- | --- | --- | --- | --- | --- | --- | --- |
| **Region** | **Peak MNI coordinate**  **(x, y, z)** | **SDM-Z**  **value** | ***p* value** |  | **No. of voxels** | **Breakdown (No. of voxels)** | |
| ***EOS vs. HCs (EOS＞HCs)*** | | | | | | |  |
| Left middle frontal gyrus, BA 46 | -38, 48, 4 | 5.18 | 1.19e-7 |  | 884 | Left inferior frontal gyrus, triangular part, BA 45 (175)  Left middle frontal gyrus, BA 46 (114)  Left anterior thalamic projections (112)  Left inferior frontal gyrus, orbital part, BA 47 (102)  Left middle frontal gyrus, orbital part, BA 46 (86)  Left middle frontal gyrus, orbital part, BA 47 (69)  Left middle frontal gyrus, BA 10 (63)  Left inferior network, inferior fronto-occipital fasciculus (50)  Left inferior frontal gyrus, orbital part, BA 46 (19)  Left inferior frontal gyrus, triangular part, BA 46 (18)  Left middle frontal gyrus, BA 47 (16)  Left inferior frontal gyrus, triangular part, BA 47 (9)  Left middle frontal gyrus, orbital part, BA 10 (9)  Left inferior frontal gyrus, orbital part, BA 45 (8)  Left middle frontal gyrus, BA 45 (8)  Left middle frontal gyrus, orbital part (6)  Corpus callosum (3)  Left inferior frontal gyrus, triangular part (3)  Left superior frontal gyrus, dorsolateral, BA 10 (2)  Left middle frontal gyrus, BA 11 (2)  Left superior frontal gyrus, dorsolateral, BA 47 (1)  Left superior frontal gyrus, dorsolateral, BA 11 (1)  Left striatum (1)  Left superior frontal gyrus, orbital part, BA 11 (1)  Left middle frontal gyrus, orbital part, BA 45 (1)  Left middle frontal gyrus (1)  Left superior frontal gyrus, orbital part, BA 47 (1)  (undefined) (2)  (undefined), BA 47 (1) | |
| Right caudate nucleus | 12, 4, 18 | 4.88 | 5.36e-7 |  | 660 | Right anterior thalamic projections (313)  Right caudate nucleus (211)  Right caudate nucleus, BA 25 (46)  Corpus callosum (38)  Right striatum (2)  (undefined) (50) | |
| Corpus callosum | 8, 18, 22 | 3.20 | 6.81e-4 |  | 29 | Corpus callosum (13)  Right anterior cingulate / paracingulate gyri (6)  Right median network, cingulum (6)  Right anterior cingulate / paracingulate gyri, BA 24 (4) | |
| ***EOS vs. HCs (EOS＜HCs)*** | | | | | | | |
| Left superior temporal gyrus, BA 48 | -58, -12, 0 | -2.78 | 2.70e-3 |  | 26 | Corpus callosum (16)  Left superior temporal gyrus, BA 48(8)  Left superior temporal gyrus, BA 22 (2) | |

**Abbreviations:** HCs, healthy controls; EOS, early-onset schizophrenia; MNI, Montreal Neurological Institute; SDM, seed-based *d* mapping; BA, Brodmann area.

**Table S7 GMV differences between EOS patients compared to HCs (excluding studies of uncorrected for statistics).**

| **Local Maximum** |  |  |  |  | **Cluster** | |
| --- | --- | --- | --- | --- | --- | --- |
| **Region** | **Peak MNI coordinate**  **(x, y, z)** | **SDM-Z**  **value** | ***p* value** |  | **No. of voxels** | **Breakdown (No. of voxels)** |
| ***EOS vs. HCs (EOS＜HCs)*** | | | | | | |
| Right superior temporal gyrus, BA 48 | 60, -18, 8 | -3.13 | 8.91e-4 |  | 145 | Corpus callosum (51)  Right superior temporal gyrus, BA 22 (35)  Right superior temporal gyrus, BA 48 (34)  Right rolandic operculum, BA 48 (13)  Right superior temporal gyrus (6)  Right rolandic operculum (4)  Right heschl gyrus, BA 48 (2) |

**Abbreviations:** HCs, healthy controls; EOS, early-onset schizophrenia; MNI, Montreal Neurological Institute; SDM, seed-based *d* mapping; BA, Brodmann area.

**Table S8 Resting-state functional brain activity differences between first-episode EOS patients compared to HCs.**

| **Local Maximum** |  |  |  |  | **Cluster** | |
| --- | --- | --- | --- | --- | --- | --- |
| **Region** | **Peak MNI coordinate**  **(x, y, z)** | **SDM-Z**  **value** | ***p* value** |  | **No. of voxels** | **Breakdown (No. of voxels)** |
| ***EOS vs. HCs (EOS＞HCs)*** | | | | | | |
| Left inferior frontal gyrus, triangular part, BA 45 | -40, 42, 2 | 3.57 | 1.78e-4 |  | 550 | Left inferior frontal gyrus, triangular part, BA 45 (97)  Left anterior thalamic projections (82)  Left middle frontal gyrus, BA 46 (73)  Left middle frontal gyrus, orbital part, BA 46 (68)  Left middle frontal gyrus, orbital part, BA 47 (61)  Left inferior frontal gyrus, orbital part, BA 47 (49)  Left middle frontal gyrus, BA 10 (36)  Left middle frontal gyrus, BA 47 (16)  Left inferior network, inferior fronto-occipital fasciculus (15)  Left inferior frontal gyrus, orbital part, BA 46 (13)  Left inferior frontal gyrus, triangular part, BA 46 (12)  Left inferior frontal gyrus, triangular part, BA 47 (9)  Left inferior frontal gyrus, orbital part, BA 45 (5)  Left middle frontal gyrus, BA 45 (4)  Left middle frontal gyrus, orbital part, BA 10 (3)  Left middle frontal gyrus, orbital part (3)  Left superior frontal gyrus, dorsolateral, BA 10 (1)  Left superior frontal gyrus, dorsolateral, BA 11 (1)  Left inferior frontal gyrus, triangular part (1)  Left middle frontal gyrus, orbital part, BA 45 |
| ***EOS vs. HCs (EOS＜HCs)*** | | | | | | |
| Left postcentral gyrus, BA 43 | -60, -16, 30 | -2.78 | 2.71e-3 |  | 13 | Left postcentral gyrus, BA 43 (10)  Left postcentral gyrus, BA 48 (3) |

**Abbreviations:** HCs, healthy controls; EOS, early-onset schizophrenia; MNI, Montreal Neurological Institute; SDM, seed-based *d* mapping; BA, Brodmann area.

**Table S9 Resting-state functional brain activity differences between drug-naive EOS patients compared to HCs.**

| **Local Maximum** |  |  |  |  | **Cluster** | |
| --- | --- | --- | --- | --- | --- | --- |
| **Region** | **Peak MNI coordinate**  **(x, y, z)** | **SDM-Z**  **value** | ***p* value** |  | **No. of voxels** | **Breakdown (No. of voxels)** |
| ***EOS vs. HCs (EOS＞HCs)*** | | | | | | |
| Left middle frontal gyrus, BA 46 | -40, 48, 8 | 3.63 | 1.44e-4 |  | 615 | Left inferior frontal gyrus, triangular part, BA 45 (138)  Left middle frontal gyrus, BA 46 (89)  Left anterior thalamic projections (68)  Left middle frontal gyrus, orbital part, BA 46 (67)  Left inferior frontal gyrus, orbital part, BA 47 (60)  Left middle frontal gyrus, orbital part, BA 47 (59)  Left middle frontal gyrus, BA 10 (40)  Left inferior frontal gyrus, orbital part, BA 46 (17)  Left inferior frontal gyrus, triangular part, BA 46 (16)  Left inferior network, inferior fronto-occipital fasciculus (16)  Left middle frontal gyrus, BA 47 (16)  Left inferior frontal gyrus, triangular part, BA 47 (8)  Left inferior frontal gyrus, orbital part, BA 45 (6)  Left middle frontal gyrus, BA 45 (5)  Left middle frontal gyrus, orbital part (3)  Left middle frontal gyrus, orbital part, BA 10 (2)  Left superior frontal gyrus, dorsolateral, BA 10 (1)  Left inferior frontal gyrus, triangular part (1)  Corpus callosum (1)  Left middle frontal gyrus, orbital part, BA 45 (1)  (undefined) (1) |
| Right caudate nucleus | 16, 22, 4 | 3.50 | 2.37e-4 |  | 551 | Right anterior thalamic projections (261)  Right caudate nucleus (186)  Right caudate nucleus, BA 25 (42)  Corpus callosum (27)  (undefined) (35) |

**Abbreviations:** HCs, healthy controls; EOS, early-onset schizophrenia; MNI, Montreal Neurological Institute; SDM, seed-based *d* mapping; BA, Brodmann area.

**Figure**
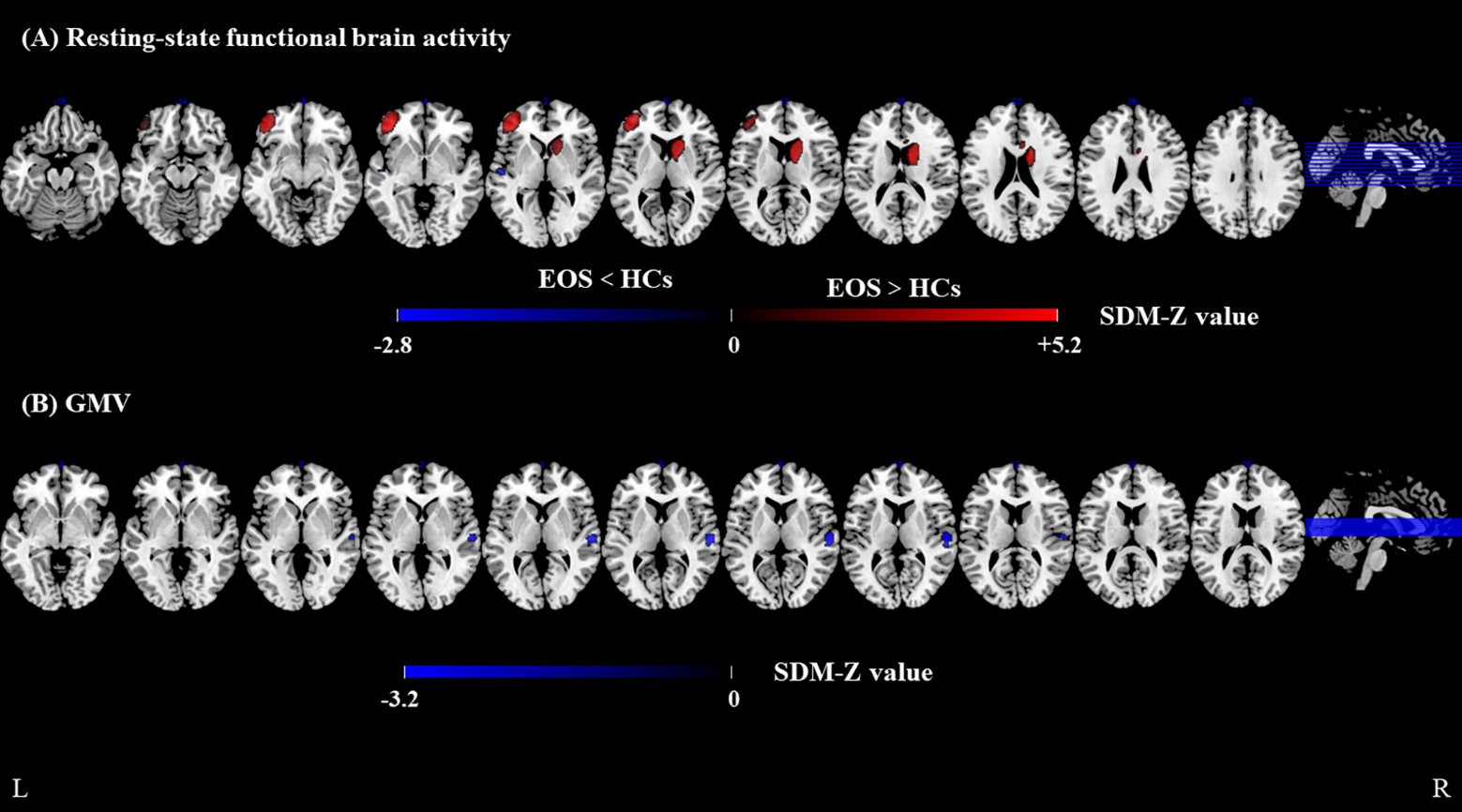
**S1**

**Figure**
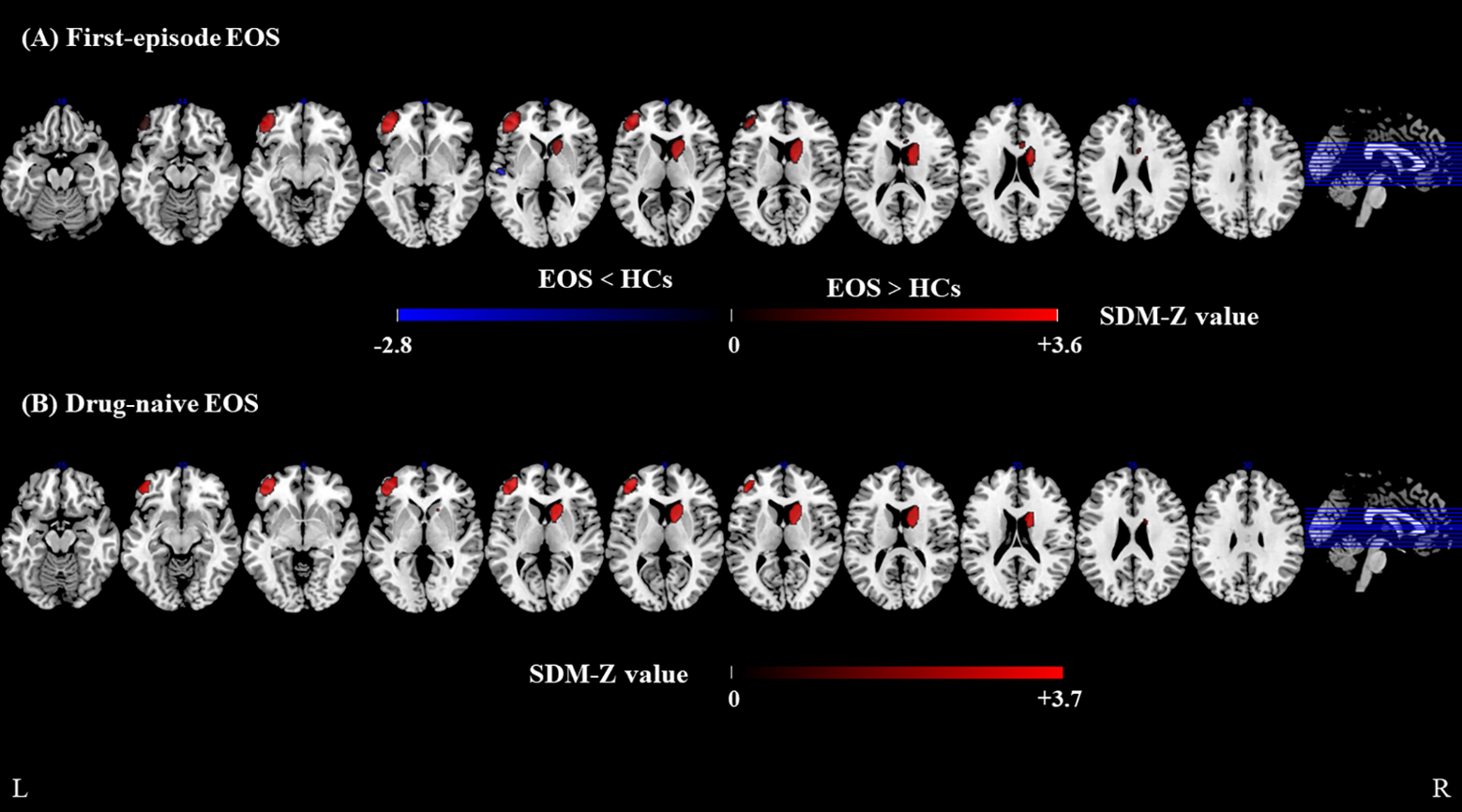
**S2**

**Figure S1. Subgroup analyses after excluding studies of uncorrected for statistics.** (A) resting-state regional functional activity difference between EOS and HCs, (B) GMV difference between EOS and HCs. Regions with decreased resting-state regional functional activity or GMV are displayed in blue, and regions with increased resting-state regional functional activity or GMV are displayed in red. The color bar indicates the maximum and minimum SDM-Z values. Abbreviations: EOS = early-onset schizophrenia; HCs = healthy controls; SDM = Seed-based *d* mapping; GMV = gray matter volume.

**Figure S2. Subgroup analyses for studies of resting-state regional functional activity.** (A) resting-state regional functional activity difference between first-episode EOS and HCs, (B) resting-state regional functional activity difference between drug-naive EOS and HCs. Regions with decreased resting-state regional functional activity or GMV are displayed in blue, and regions with increased resting-state regional functional activity or GMV are displayed in red. The color bar indicates the maximum and minimum SDM-Z values. Abbreviations: EOS = early-onset schizophrenia; HCs = healthy controls; SDM = Seed-based *d* mapping.

**REFERENCES**

1. Jiang SA, Zhou B, Liao YH, Liu WQ, Tian CL, Chen XG, et al. Primary Study of Resting State Functional Magnetic Resonance Imaging in Early Onset Schizophrenia Using Reho. *J Cent South Univ (Med Sci)* (2010) 35(9):947-51. Epub 2010/09/28. doi: 10.3969/j.issn.1672-7347.2010.09.008.

2. Zheng J, Zhang Y, Guo X, Duan X, Zhang J, Zhao J, et al. Disrupted Amplitude of Low-Frequency Fluctuations in Antipsychotic-Naïve Adolescents with Early-Onset Schizophrenia. *Psychiatry research Neuroimaging* (2016) 249:20-6. Epub 2016/03/24. doi: 10.1016/j.pscychresns.2015.11.006.

3. Xiong YB, Ren Y, Cui XH, Xu WY, Sun XL, Yang H. Resting State Fmri Study of Amplitude of Low-Frequency Fluctuation in Early Onset Schizophrenia. *Chin J Nerv Ment Dis* (2016) 42(5):272-6. Epub 2016/07/28. doi: 10.3969/j.issn.1002-0152.2016.05.004.

4. Lü D, Shao RR, Liang YH, Xia YH, Guo SQ. [Fractional Amplitude of Low-Frequency Fluctuations in Childhood and Adolescence-Onset Schizophrenia: A Resting State Fmri Study]. *Zhonghua yi xue za zhi* (2016) 96(43):3479-84. Epub 2016/12/03. doi: 10.3760/cma.j.issn.0376-2491.2016.43.008.

5. Liang Y, Shao R, Zhang Z, Li X, Zhou L, Guo S. Amplitude of Low-Frequency Fluctuations in Childhood-Onset Schizophrenia with or without Obsessive-Compulsive Symptoms: A Resting-State Functional Magnetic Resonance Imaging Study. *Archives of medical science : AMS* (2019) 15(1):126-33. Epub 2019/01/31. doi: 10.5114/aoms.2018.73422.

6. Pei QL, Zhang HS, Wang B, Wang X, Zhao QJ. Study of Intracerebral Loop in Patients with Schizophrenia without Early Onset of Schizophrenia by Resting State Functional Magnetic Resonance Imaging. *Chin J CT MR* (2018) 16(7):68-71. Epub 2018/11/28. doi: 10.3969/j.issn.1672-5131.2018.07.021.

7. Wang S, Zhang Y, Lv L, Wu R, Fan X, Zhao J, et al. Abnormal Regional Homogeneity as a Potential Imaging Biomarker for Adolescent-Onset Schizophrenia: A Resting-State Fmri Study and Support Vector Machine Analysis. *Schizophrenia research* (2018) 192:179-84. Epub 2017/06/08. doi: 10.1016/j.schres.2017.05.038.

8. Li YL, Li K, Wang B, Liang YH, Xia YH, Li YL, et al. Study of Resting-State Mri in First-Episode Childhood and Adolescence—Onset Schizophrenia with and without Auditory Hallucinatiolls. *Chin J Nerv Ment Dis* (2019) 45(8):454-9. Epub 2019/10/30. doi: 10.3969/j.issn.1002-0152.2019.08.002.

9. Lyu H, Jiao J, Feng G, Wang X, Sun B, Zhao Z, et al. Abnormal Causal Connectivity of Left Superior Temporal Gyrus in Drug-Naïve First- Episode Adolescent-Onset Schizophrenia: A Resting-State Fmri Study. *Psychiatry research Neuroimaging* (2021) 315:111330. Epub 2021/07/20. doi: 10.1016/j.pscychresns.2021.111330.

10. Li YL, Li YD, Zhang H, Gao ZT, Xia YH, Liang YH, et al. [Relationship between Auditory Hallucination and Regional Homogeneity of Functional Magnetic Resonance Imaging in First-Episode Childhood and Adolescence-Onset Schizophrenia]. *Zhonghua yi xue za zhi* (2021) 101(24):1915-20. Epub 2021/10/09. doi: 10.3760/cma.j.cn112137-20201126-03195.

11. Yang Y, Sun Y, Zhang Y, Jin X, Li Z, Ding M, et al. Abnormal Patterns of Regional Homogeneity and Functional Connectivity across the Adolescent First-Episode, Adult First-Episode and Adult Chronic Schizophrenia. *NeuroImage Clinical* (2022) 36:103198. Epub 2022/09/19. doi: 10.1016/j.nicl.2022.103198.

12. Liang Y, Shao R, Xia Y, Li Y, Guo S. Investigating Amplitude of Low-Frequency Fluctuation and Possible Links with Cognitive Impairment in Childhood and Adolescence Onset Schizophrenia: A Correlation Study. *Frontiers in psychiatry* (2024) 15:1288955. Epub 2024/03/01. doi: 10.3389/fpsyt.2024.1288955.

13. Douaud G, Smith S, Jenkinson M, Behrens T, Johansen-Berg H, Vickers J, et al. Anatomically Related Grey and White Matter Abnormalities in Adolescent-Onset Schizophrenia. *Brain : a journal of neurology* (2007) 130(Pt 9):2375-86. Epub 2007/08/19. doi: 10.1093/brain/awm184.

14. Pagsberg AK, Baaré WF, Raabjerg Christensen AM, Fagerlund B, Hansen MB, Labianca J, et al. Structural Brain Abnormalities in Early Onset First-Episode Psychosis. *Journal of neural transmission (Vienna, Austria : 1996)* (2007) 114(4):489-98. Epub 2006/10/07. doi: 10.1007/s00702-006-0573-8.

15. Yoshihara Y, Sugihara G, Matsumoto H, Suckling J, Nishimura K, Toyoda T, et al. Voxel-Based Structural Magnetic Resonance Imaging (Mri) Study of Patients with Early Onset Schizophrenia. *Annals of general psychiatry* (2008) 7:25. Epub 2008/12/24. doi: 10.1186/1744-859x-7-25.

16. Janssen J, Reig S, Parellada M, Moreno D, Graell M, Fraguas D, et al. Regional Gray Matter Volume Deficits in Adolescents with First-Episode Psychosis. *Journal of the American Academy of Child and Adolescent Psychiatry* (2008) 47(11):1311-20. Epub 2008/10/02. doi: 10.1097/CHI.0b013e318184ff48.

17. Wei QL, Kang Z, Wu XL, Zhang JB, Li LJ, Luo ZX, et al. Morphological Abnormities in Early-Onset and Late-Onset Schizophrenia Patients. *J SUN Yat⁃sen Univ (Med Sci)* (2011) 32(4):527-30. Epub 2011/12/06.

18. Tang J, Liao Y, Zhou B, Tan C, Liu W, Wang D, et al. Decrease in Temporal Gyrus Gray Matter Volume in First-Episode, Early Onset Schizophrenia: An Mri Study. *PloS one* (2012) 7(7):e40247. Epub 2012/07/18. doi: 10.1371/journal.pone.0040247.

19. Fang JC, Li DM, Chen XD, Nie BB, Liu H, Wang JJ, et al. Morphological Abnormities in Early-Onset and Late-Onset Schizophrenia Patients. *Chinese Journal of Clinical Psychology* (2015) 23(5):839-42, 90. doi: 10.16128/j.cnki.1005-3611.2015.05.019.

20. Zhang Y, Zheng J, Fan X, Guo X, Guo W, Yang G, et al. Dysfunctional Resting-State Connectivities of Brain Regions with Structural Deficits in Drug-Naive First-Episode Schizophrenia Adolescents. *Schizophrenia research* (2015) 168(1-2):353-9. Epub 2015/08/19. doi: 10.1016/j.schres.2015.07.031.

21. Zhang C, Wang Q, Ni P, Deng W, Li Y, Zhao L, et al. Differential Cortical Gray Matter Deficits in Adolescent- and Adult-Onset First-Episode Treatment-Naïve Patients with Schizophrenia. *Scientific reports* (2017) 7(1):10267. Epub 2017/09/02. doi: 10.1038/s41598-017-10688-1.

22. Zhang L, Wang X, Zhang Y, Yang YF, Li WW, Li BL, et al. [Cerebral Grey Matter Changes of Pre- and Post-Treatment in First-Episode Drug-Naive Adolescents Schizophrenia]. *Zhonghua yi xue za zhi* (2018) 98(37):2968-72. Epub 2018/11/06. doi: 10.3760/cma.j.issn.0376-2491.2018.37.003.

23. Castro-Fornieles J, Bargalló N, Calvo A, Arango C, Baeza I, Gonzalez-Pinto A, et al. Gray Matter Changes and Cognitive Predictors of 2-Year Follow-up Abnormalities in Early-Onset First-Episode Psychosis. *European child & adolescent psychiatry* (2018) 27(1):113-26. Epub 2017/07/15. doi: 10.1007/s00787-017-1013-z.

24. Gao ZT, Li YL, Guo SQ, Xia YH. [Brain Gray Matter Volume Alterations and Cognitive Function in First-Episode Childhood-and Adolescence-Onset Schizophrenia]. *Zhonghua yi xue za zhi* (2019) 99(45):3581-6. Epub 2019/12/13. doi: 10.3760/cma.j.issn.0376-2491.2019.45.010.

25. Shen Y, Gao X, Huang C, Luo X, Ge R. Decreased Gray Matter Volume Is Associated with Theory of Mind Deficit in Adolescents with Schizophrenia. *Brain imaging and behavior* (2022) 16(3):1441-50. Epub 2022/01/22. doi: 10.1007/s11682-021-00591-9.

26. Li Q, Liu S, Cao X, Li Z, Fan YS, Wang Y, et al. Disassociated and Concurrent Structural and Functional Abnormalities in the Drug-Naïve First-Episode Early Onset Schizophrenia. *Brain imaging and behavior* (2022) 16(4):1627-35. Epub 2022/02/19. doi: 10.1007/s11682-021-00608-3.

27. Cai J, Wei W, Zhao L, Li M, Li X, Liang S, et al. Abnormal Brain Structure Morphology in Early-Onset Schizophrenia. *Frontiers in psychiatry* (2022) 13:925204. Epub 2022/07/26. doi: 10.3389/fpsyt.2022.925204.
